# Supplementary material for: Enhanced Adsorption of Sulfonamides by Attapulgite-Doped Biochar Prepared with Calcination
Source: Molecules. 2022 Nov 21;27(22):8076. doi: 10.3390/molecules27228076 (PMC9698770; doi:10.3390/molecules27228076)
Supplement: Supplementary file 1 [file molecules-27-08076-s001.zip › molecules-2026356-supplementary.pdf]

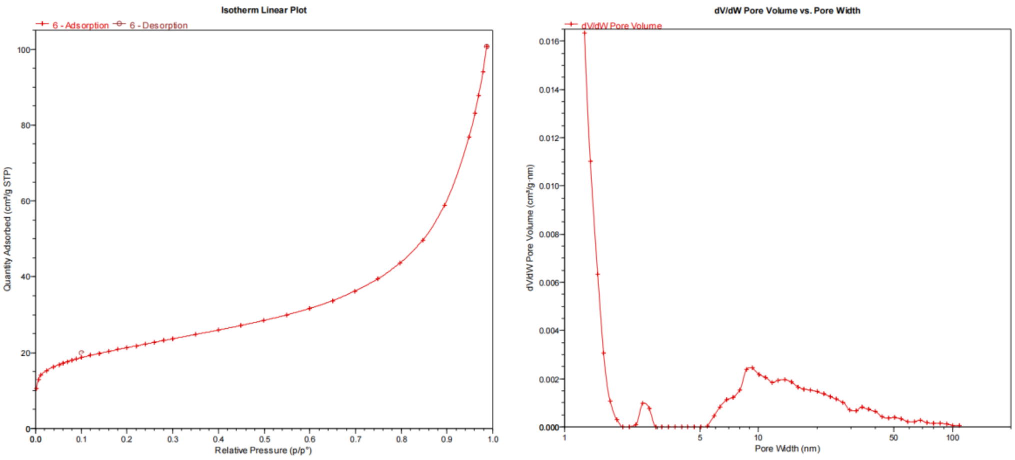

Figure S1. The adsorption-desorption isotherm and pore size distribution of ATP.

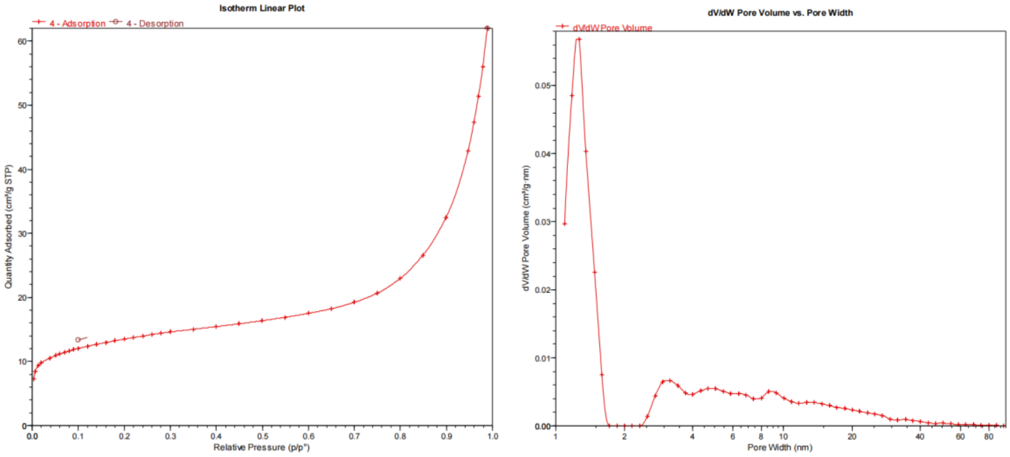

Figure S2. The adsorption-desorption isotherm and pore size distribution of BC.

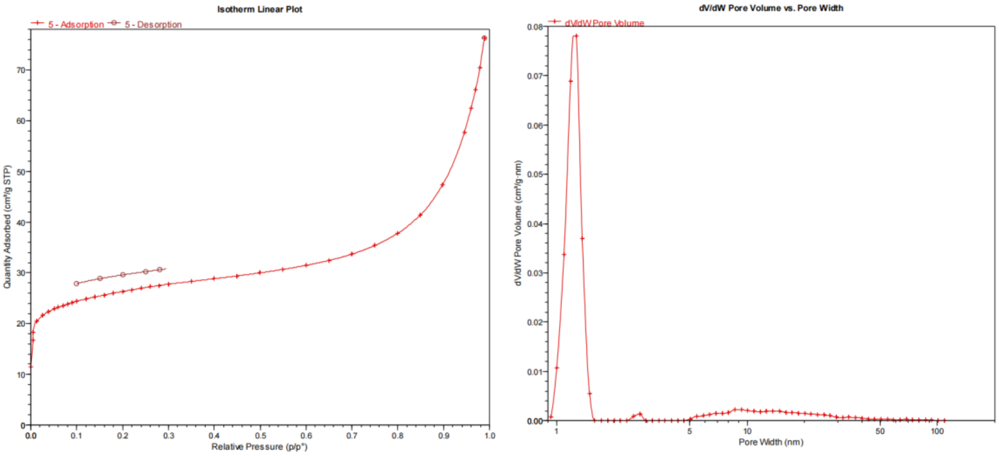

Figure S3. The adsorption-desorption isotherm and pore size distribution of ATP/BC-0.01.

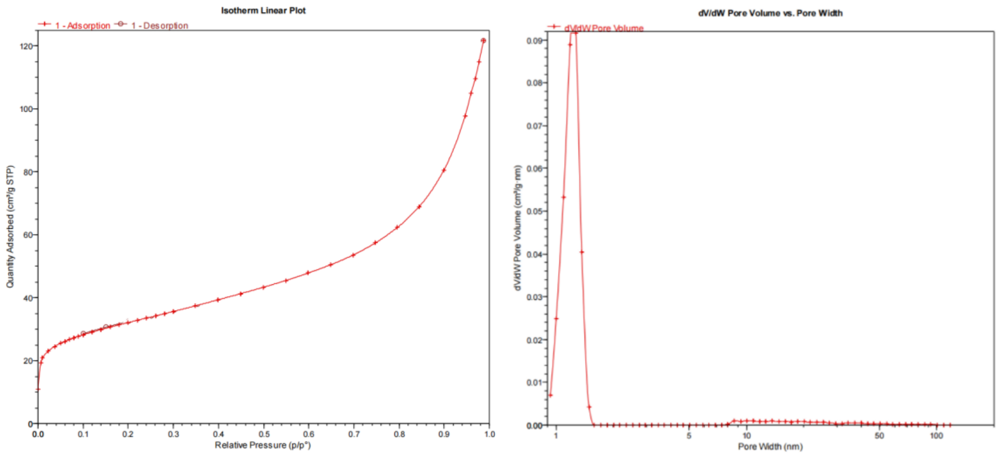

Figure S4. The adsorption-desorption isotherm and pore size distribution of ATP/BC-0.1.

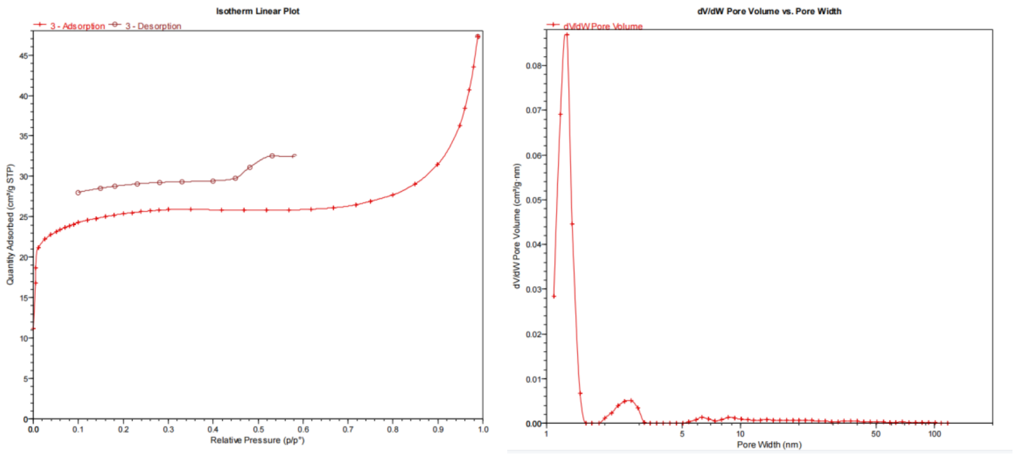

Figure S5. The adsorption-desorption isotherm and pore size distribution of ATP/BC-0.3.

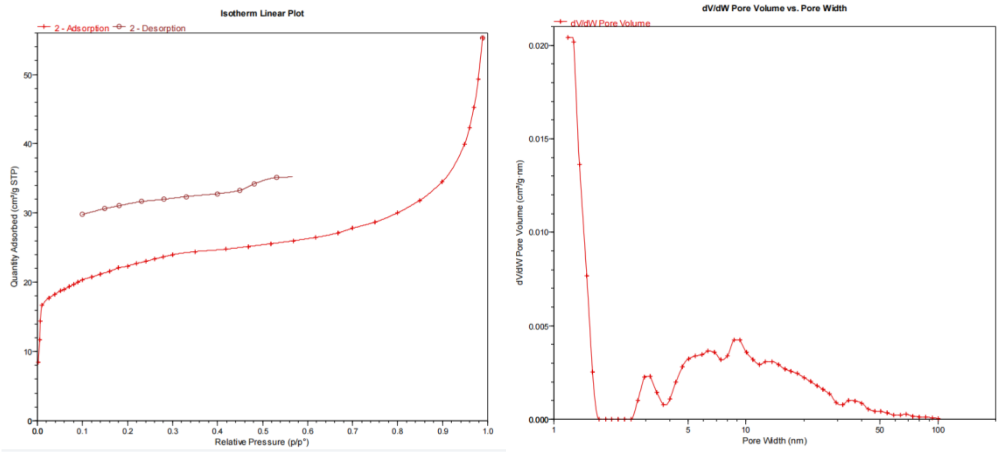

Figure S6. The adsorption-desorption isotherm and pore size distribution of ATP/BC-0.5.

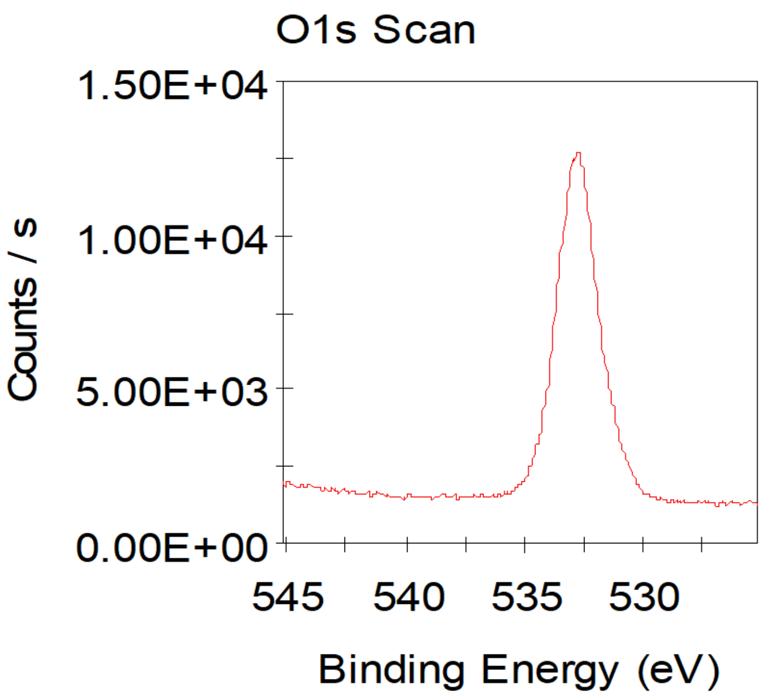

Figure S7. The XPS O1s scan of BC.

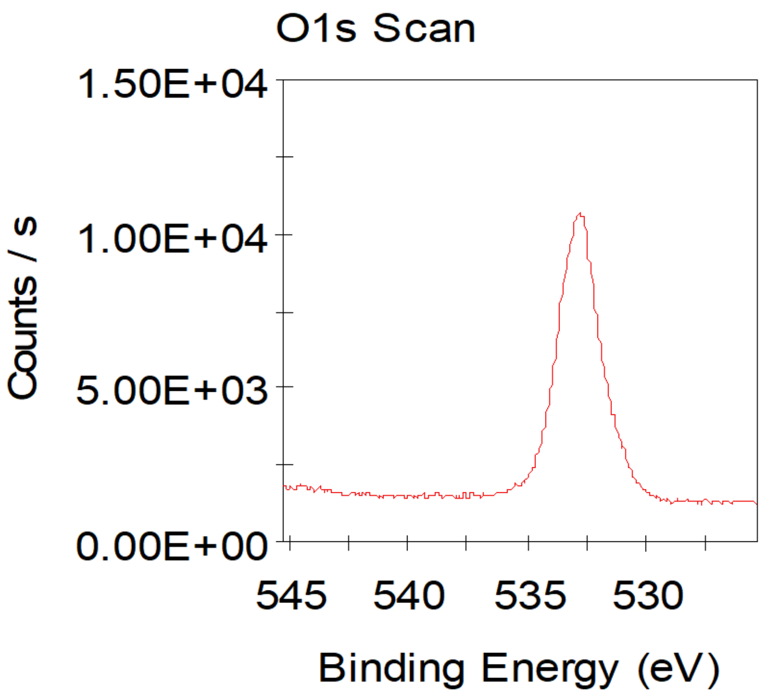

Figure S8. The XPS O1s scan of ATP/BC.
